# The TLR-NF-kB axis contributes to the monocytic inflammatory response against a virulent strain of *Lichtheimia corymbifera*, a causative agent of invasive mucormycosis

**DOI:** 10.3389/fimmu.2022.882921

**Published:** 2022-10-13

**Authors:** Dolly E. Montaño, Susann Hartung, Melissa Wich, Rida Ali, Berit Jungnickel, Marie von Lilienfeld-Toal, Kerstin Voigt

**Affiliations:** ^1^ Jena Microbial Resource Collection, Leibniz Institute for Natural Product Research and Infection Biology – Hans Knöll Institute (HKI), Jena, Germany; ^2^ Jena Microbial Resource Collection, Institute of Microbiology, Friedrich Schiller University Jena, Jena, Germany; ^3^ Infections in Hematology and Oncology, Leibniz Institute for Natural Product Research and Infection Biology – Hans Knöll Institute (HKI), Jena, Germany; ^4^ Center for Molecular Biomedicine (CMB), Friedrich Schiller University Jena, Jena, Germany; ^5^ Department of Hematology and Medical Oncology, Jena University Hospital, Jena, Germany

**Keywords:** Mucorales, mucormycosis, phagocytosis, inflammatory response, receptors, cytokines, cell damage, apoptosis

## Abstract

Invasive mucormycosis (IM) is a life-threatening infection caused by the fungal order Mucorales, its diagnosis is often delayed, and mortality rates range from 40-80% due to its rapid progression. Individuals suffering from hematological malignancies, diabetes mellitus, organ transplantations, and most recently COVID-19 are particularly susceptible to infection by Mucorales. Given the increase in the occurrence of these diseases, mucormycosis has emerged as one of the most common fungal infections in the last years. However, little is known about the host immune response to Mucorales. Therefore, we characterized the interaction among *L. corymbifera—*one of the most common causative agents of IM—and human monocytes, which are specialized phagocytes that play an instrumental role in the modulation of the inflammatory response against several pathogenic fungi. This study covered four relevant aspects of the host-pathogen interaction: i) The recognition of *L. corymbifera* by human monocytes. ii) The intracellular fate of *L. corymbifera.* iii) The inflammatory response by human monocytes against the most common causative agents of mucormycosis. iv) The main activated Pattern-Recognition Receptors (PRRs) inflammatory signaling cascades in response to *L. corymbifera*. Here, we demonstrate that *L. corymbifera* exhibits resistance to intracellular killing over 24 hours, does not germinate, and inflicts minimal damage to the host cell. Nonetheless, viable fungal spores of *L. corymbifera* induced early production of the pro-inflammatory cytokine IL-1β, and late release of TNF-α and IL-6 by human monocytes. Moreover, we revealed that IL-1β production predominantly depends on Toll-like receptors (TLRs) priming, especially *via* TLR4, while TNF-α is secreted *via* C-type lectin receptors (CTLs), and IL-6 is produced by synergistic activation of TLRs and CTLs. All these signaling pathways lead to the activation of NF-kB, a transcription factor that not only regulates the inflammatory response but also the apoptotic fate of monocytes during infection with *L. corymbifera.* Collectively, our findings provide new insights into the host-pathogen interactions, which may serve for future therapies to enhance the host inflammatory response to *L. corymbifera*.

## Introduction

During the last years, the number of mucormycosis cases has increased (1–3) along with the predisposing risk factors such as hematological malignancies, diabetes, organ transplantations, and corticosteroid therapies (3–5). Mucormycosis, an infection caused by the fungal order of Mucorales, is characterized by a fast invasion of blood vessels, thrombosis, and tissue necrosis. The outcome of this infection commonly results in high mortality rates due to the rapid destruction of tissue, difficult early diagnosis, and resistance to antifungal agents (6–8). Within the Mucorales, *Lichtheimia* species are among the most important causative agents together with *Rhizopus* and *Mucor* species (3, 5, 9). These molds are currently emerging as opportunistic fungi and exhibit devastating results in immunocompromised patients (10), but also immunocompetent patients can suffer from Farmer’s lung disease, a form of hypersensitivity pneumonitis resulting from recurrent exposure to moldy plant materials (11). After inhalation by humans, the dormant sporangiospores (here assigned as resting spores) of Mucorales encounter several interactions with the host’s adaptive and innate immune system of which just a few have been studied as drivers of pathogenesis [as reviewed by (2, 12, 13)]. Hence, we found it highly relevant to understand their interaction with the host-immune cells and the mechanism that triggers antifungal alarm systems, such as the inflammatory response.

Antifungal immunity requires strict regulation among different cellular processes to eliminate the fungus and protect the organism from collateral damages (14). In that regard, specialized proteins known as cytokines and chemokines promote a balanced communication among several cellular types, modulate phagocyte recruitment, antigen presentation, activation, and immune cell maturation as well as activation and resolution of the inflammatory response, which includes clearance of the pathogen and tissue repair (14, 15). Besides other professional phagocytes, monocytes are instrumental in the modulation of the inflammatory response against several pathogenic fungi (16–18). These immune cells circulate in the bloodstream and migrate into infected tissue attracted by chemokines, where they differentiate into macrophages or dendritic cells (16, 19, 20). Furthermore, monocytes are the main source of IL-1β in humans, a cytokine involved in the initiation of immune responses, induction of further pro-inflammatory proteins, hematopoiesis, differentiation of T_H_17 cells, development of IL-10, and a typical marker of inflammasome activation (15, 21–23). Additionally, monocytes produce TNF-α and IL-6 which are pro-inflammatory cytokines that mediate host-apoptosis and inflammation as well as trafficking of leukocytes, production of acute-phase proteins, T-cell proliferation, B-cell differentiation, and survival (24, 25).

To initiate the inflammatory response against pathogenic fungi, immune cells recognize cell-wall constituents known as Pathogen-Associated Molecular Patterns (PAMPs), such as cell-wall proteins, glucans, chitin, and lipids, or endogenous signals known as damage-associated molecular patterns (DAMPs) (26, 27). This fungal recognition is mediated by Pattern-Recognition Receptors (PRRs), which are specialized cell-surface receptors triggering pathogen-killing and inflammatory signaling cascades upon stimulation (14, 15, 27). Furthermore, PRRs are classified into several families including Toll-like receptors (TLRs), C-type lectin receptors (CLRs), and NOD-like receptors (NLRs) which modulate several inflammatory signaling pathways resulting in cytokine production (28, 29). For this reason, unraveling the recognition mechanism and the modulation of the inflammatory response against *Lichtheimia corymbifera* could provide novel insights for diagnostics biomarkers and therapies. Therefore, in this study we focused on three relevant aspects of the host response against this fungus: i) characterization of *L. corymbifera* recognition by human monocytes. ii) Comparing the pro-inflammatory cytokine profile among the most causative agents of mucormycosis. iii) Determining predominant PRRs axes that prime the inflammatory response against *L. corymbifera* and its connection to the innate apoptotic immunity. We did not further characterize the host-pathogen interaction with *Rhizopus* and *Mucor* species since this subject has been previously described (30–34).

## Materials and methods

### Ethics statement

Human blood was obtained from the Institute for Transfusion Medicine, Jena University Hospital, after written informed consent of the healthy donors in accordance with ethics committee approval 4357-03/15 and with the Declaration of Helsinki 1975, as revised in 2008. Mice experiments were approved by Thueringer Landesamt für Verbraucherschutz (breeding license 02-052/16).

### Fungal strains, cultivation, and spore treatments

The most virulent strains of *L. corymbifera* JMRC: FSU:009682 (original strain nos.: CBS 429.75, ATCC 46771), and the least virulent JMRC: FSU:0010164, *Mucor lusitanicus* (formerly: *M. circinelloides* f. *lusitanicus)* JMRC: FSU:005859 (original strain no.: CBS 277.49), and *Rhizopus arrhizus* var. *arrhizus* (formerly: *R. delemar*) JMRC: FSU:008743 (original strain no.: 99-880) were grown in Potato Dextrose Agar for seven days at 37° C. Spores were harvested by using sterile phosphate-buffered saline (PBS, 137 mM NaCl, 10 mM Na_2_HPO_4_, 2.7 mM KCl, 1.76 mM KH_2_PO_4_, pH 7.4). To inactivate spores, 4% formaldehyde (PFA) was used for 30 min at a concentration of 1x10^7^ spores/mL. Finally, spores were counted in a Thoma hemocytometer and adjusted to the desired concentration in RPMI 1640 supplemented with fetal bovine serum (FBS) (Gibco).

### Purification, cultivation, and infection of human cells

Peripheral Blood Mononuclear Cells (PBMC) were isolated from healthy donors’ buffy coats by a density gradient centrifugation using Histopaque-1077 (Sigma Aldrich). Consequently, human monocytes were positively selected from PBMC using CD14 MicroBeads coupled to a magnetic cell sorting system (Miltenyi Biotec). Isolated human monocytes were seeded into twenty-four-well plates at a density of 1x10^6^ cells per well and cultured in RPMI-1640 (Lonza) supplemented with 10% heat-inactivated FBS (Gibco) for two hours at 37 °C and 5% CO_2_. Resting monocytes were infected with *L. corymbifera* spores at a multiplicity of infection (MOI) of 5 at the corresponding times.

### Phagocytosis quantification by flow cytometry

The method was adapted from Hartung et al. (Cytometry A. 2019 Mar;95(3):332-338). Briefly, erythrocytes were depleted from healthy donors’ blood by osmotic shock using an Erythrocyte Lysis Buffer (0.15 M NH_4_Cl; 10 mM NH_4_Cl; 0.1 mM EDTA). Human leukocytes were seeded in a 24-well plate at a density of 1x10^6^ cells per well and cultured in RPMI 1640 (Lonza) supplemented with 10% heat-inactivated FBS (Gibco). The obtained leukocytes were incubated with fluorescein isothiocyanate (FITC)-labeled (100µg/mL) spores at MOI 5 for 30, 60, and 120 minutes at 37 °C and 5% CO_2_. Consequently, leukocytes were harvested with PBS-2mM EDTA and stained with anti-CD45-BUV395 (Biosciences), anti-CD15-BV605 (Biolegend), anti-CD14-PECy7 (Biolegend), and anti-FITC-APC (Invitrogen) for 20 minutes at room temperature (RT). Phagocytosis was assessed by using an LSR Fortessa (Becton Dickinson) flow cytometer and data were analyzed with FlowJo v10.6.2 software.

### Cell viability analysis

Isolated CD14+ monocytes were incubated with *L. corymbifera* spores over 24 hours in a 24-well plate with RPMI-FBS supplemented medium at 37°C, 5% CO_2_. Non-phagocytosed spores were stained with Calcofluor-white (CFW) (10 mg/mL, Sigma) for 20 min. After harvesting the cells with PBS-2mM EDTA, the monocytes were lysed with radioimmunoprecipitation assay buffer (RIPA) (200 mM NaCl; 50 mM Tris-HCl, 4 mM EDTA, 1% Deoxychocolate, 1% Triton X-100 and 0.1% w/v SDS) for 40 min on ice. Phagocytosed and adherent spores were centrifuged at 3000 rpm, treated with DNAases (500 mg/mL) for 15 minutes at 37°C, and stained with Propidium Iodide (PI) (Biolegend) for viability measurements. Monocytes were assessed in a separate sample and stained with the Fixable Viability Dye - eFluor™ 780 APC-Cy7 (Thermo Scientific). Viability was quantified by using an LSR Fortessa (Becton Dickinson) flow cytometer and the data were analyzed with FlowJo v10.6.2 software.

### Fluorescence microscopic monitoring of the intracellular fate of *L. corymbifera* in monocytes

Human monocytes (1x10^6^/well) on a 12-mm-diameter coverslip were infected with FITC-labeled spores at MOI 5 for 2 hours. Non-phagocytosed spores were removed with PBS. To monitor the diameter of internalized spores, the cells were incubated for 1, 2, 4, 6, and 16 hours in RPMI medium (Lonza) supplemented with 10% heat-inactivated FBS (Gibco) at 37 °C and 5% CO2. Cells were carefully washed with PBS and counterstained with CFW (100 µg/mL, Thermo Scientific) for 20 minutes at RT. The interaction between monocytes and spores was fixed with 4% formaldehyde for 10 minutes and ProLong Gold Antifade Mountant (Thermo Scientific) was used for microscopy preparations. Microscopic observations were performed using a Zeiss LSM 780 confocal microscope, and the diameter of the internalized spores was measured by ImageJ software.

### Inhibition of Pattern Recognition Receptors

Human monocytes were positively selected by using CD14 microbeads coupled to a magnetic cell sorting system (Miltenyi Biotec). The isolated cells were incubated in a 24-well plate at 37 °C and 5% CO_2_, and stimulated for three hours with specific inhibitors reported in previous studies: 5 µM Interleukin-1 Receptor-Associated Kinase (IRAK) inhibitor (CAS 509093-47-4, Merck), 5 µM Spleen tyrosine kinase (Syk) inhibitor (ER 27319 maleate, R&D systems), 10 µM NF-KB inhibitor (Bay11-7082, *In vivo*gen), 10 µg/mL human TLR2 Neutralizing antibody (Ab) (PAb-hTLR2, *In vivo*gen), and 5 µM TLR4 inhibitor (CLI-095, *In vivo*gen) (35–38). Inhibition of IRAK, Syk, and the nuclear factor kappa-light-chain-enhancer of activated B-cells (NF-κB) was performed at a density of 1x10^6^ cells/well in 1 mL of RPMI medium supplemented with 10% v/v FBS, while TLRs at a density of 5x10^5^ cells/well in 0,5 mL of total volume. Subsequently, the monocytes were infected with the mucoralean fungi at MOI 5 for sixteen hours. Then, the supernatants were collected and the secreted cytokines were determined by ELISA MAX™ Deluxe kit (Biolegend). PAM3CSKA (*In vivo*Gen) and ultrapure Lipopolysaccharide (LPS) (*In vivo*Gen) were used as agonists for TLR axis activation, while Mannan (Sigma Aldrich) was used for CTL activation.

### Cell damage and cytokine measurement

Isolated monocytes were cultured in a 24-well plate at a density of 1x10^6^ per well and incubated with freshly harvested spores at MOI 5 for 6 and 16 hours in 1mL of RPMI (Lonza) supplemented with 10% heat-inactivated FBS (Gibco) at 37 °C and 5% CO_2_. The percentage of damage in monocyte was measured by Lactate Dehydrogenase (LDH) activity following instructions from the cytotoxic detection kit plus LDH (Roche), whereas human IL-1β, TNF-α, IL-6, and IL-10 production were assessed by using ELISA MAX™ Deluxe kit according to the manufacturer’ instructions (Biolegend).

### Induction and analysis of apoptosis in human monocytes

First, monocytes were infected for one hour as previously described. To induce apoptosis, monocytes were treated with staurosporine (STS) to a final concentration of 3.5 µM (Applichem) for three hours. Subsequently, cells were harvested and transferred into a cone-bottom 96-well plate and resuspended in PBS containing calcium and magnesium. To differentiate apoptotic stages and necrosis, monocytes were stained with 1:40 Annexin V conjugated with Pacific Blue (Invitrogen) and 1:2000 eBioscience™ Fixable Viability Dye eFluor™ 780 (Invitrogen) for 20 min at RT. Cells were analyzed using an LSR Fortessa BD flow cytometer and percentages of apoptosis were calculated with FlowJo v10.6.2 software.

### Detection of Caspase 3 and BCL-2 by western blotting

Human monocytes were lysed using RIPA buffer (see 1.5) for 40 minutes to obtain whole-cell proteins. 50 µg of total proteins were run on an SDS PAGE gel, transferred to a polyvinylidene difluoride (PVDF) blotting membrane, and incubated with human Caspase-3 mouse mAb (3G2, Cell signaling technology) overnight, and BCL-2 mouse mAb (#15071, Cell signaling technology) for 16 hours. A goat anti-mouse horseradish peroxidase (HRP, Abcam) was then incubated at RT for one hour, followed by incubation with Immobilon Western HRP Substrate (Millipore), and detection by the chemiluminescence reader Octopus QPLEX from Dye AGNOSTICS NH.

### Murine Bone Marrow-Derived Monocytes co-infection

Bone marrow from the femur of MyD88 ^-/-^ mice was isolated and differentiated into BMDMs according to (39). 7x10^5^ differentiated monocytes were cultivated in RPMI-1640 supplemented with 10% heat-inactivated FBS in a 24-well plate and infected with 7-days old, harvested spores at MOI 5 for 16 hours at 37°C and 5% CO2. Post-infection, the supernatant was collected and the IL-1β production was assessed by using ELISA MAX™ Deluxe kit according to the manufacturer’s instructions (Biolegend).

### Statistical analysis

D’Agostino & Pearson test was performed to determine normal distribution for samples with n ≥6, and Shapiro- Wilk test for n< 6. Values normally distributed were analyzed by using a one-way ANOVA with Sidak’s multiple comparisons test, and by Krustal-Wallis for non-normally distributed data, while differences among two different groups were assessed by paired t-test. All tests were performed in GraphPad Prism software version 6. A *p*-value <0.05 was considered significant and marked in graphs with the following symbols: *p, 0.05, **p, 0.01, and ***p, 0.001.

## Results

### *L. corymbifera* spores are efficiently recognized by human monocytes and neutrophils

**Figure 1:**
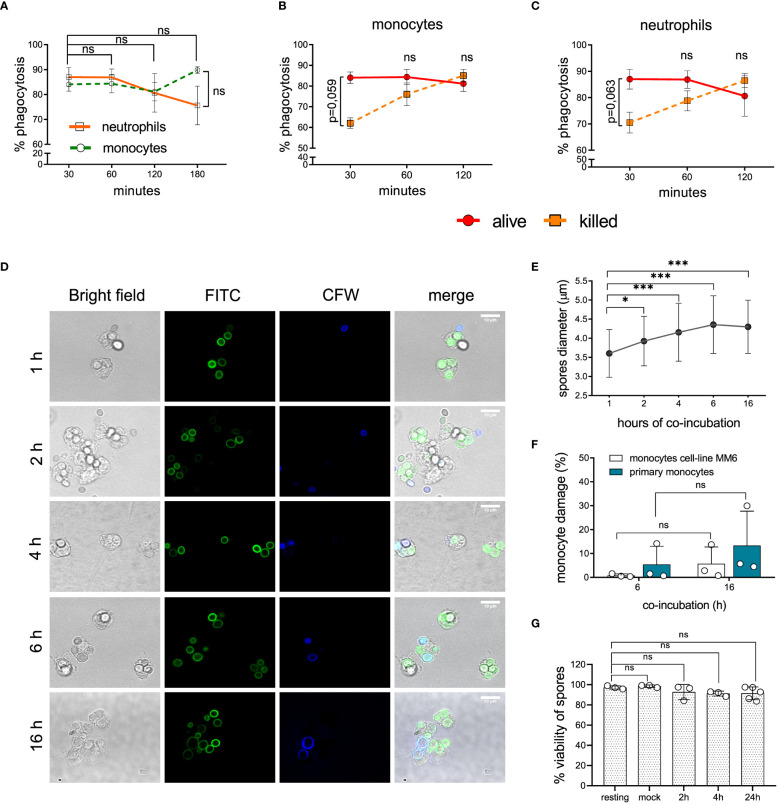
Recognition and intracellular fate of *L. corymbifera* dormant sporangiospores, here designated as resting spores. **(A)** Phagocytosis of resting spores by primary monocytes and neutrophils. **(B, C)** Phagocytosis of alive and killed fungal spores by monocytes and neutrophils. **(D)** Intracellular growth of *L. corymbifera* after phagocytosis by primary human monocytes. Fungal spores were pre-stained with FITC and counterstained with calcofluor white (CFW) after phagocytosis to differentiate attached spores (blue) from internalized spores (green). **(E)** Diameter quantification of internalized spores (n=80/experiment). **(F)** Percentage of monocytes’ LDH release after infection with *L. corymbifera*. **(G)** Viability of phagocytosed spores. Symbols and bars represent the mean ± SD of at least three independent experiments. Differences among groups were assessed by unpaired t test or one-way ANOVA. *p < 0,05; ***p <0,001. ns indicates not significant.

Phagocytosis evasion mechanisms contribute to fungal pathogenicity and prevent optimal host-immune response (40). Monocytes and neutrophils range among the professional phagocytes controlling the early stages of fungal infections (16, 41). Therefore, we evaluated the recognition efficacy of *L. corymbifera* spores by human monocytes and neutrophils, as well as the intracellular fate in human monocytes (**Figure 1**, **S2**). During the initial thirty minutes of infection (**Figure 1A**), we found (84,08 ± 5,48) phagocytosis percentage of *L. corymbifera* resting spores (alive) by human monocytes and (87,03 ± 7,54) percentage by neutrophils. Moreover, we killed *L. corymbifera* spores with formaldehyde to determine if fungal viability contribute to the recognition of *L. corymbifera*. Although phagocytosis was initially slightly decreased with killed spores (**Figures 1B, C**), we did not find significant differences compared to viable spores (**Figure 1B, C**). Independently of the treatment, all spores were similarly recognized at 120 minutes of infection and reach a phagocytic saturated stage (**Figure 1B, C**). These results indicate that monocytes and neutrophils efficiently recognize *L. corymbifera* spores.

### *L. corymbifera* spores exhibit long term survival within human monocytes

After engulfment, some pathogenic fungi use diverse mechanisms to avoid intracellular killing including inhibition of phagolysosome formation or lytic escape. This might result in further spread of the fungus and a worse outcome of the infection (30, 42). To evaluate the intracellular fate of *L. corymbifera*, we monitored its growth after phagocytosis by human monocytes (**Figure 1D**). Hence, we incubated FITC-labeled spores of *L. corymbifera* with monocytes, followed by counterstaining with CFW to distinguish adherent spores (blue) from internalized spores (green), which increased their diameter over sixteen hours of infection, but did not germinate (**Figure 1E**), contrary to viable spores cultured under the same conditions but in absence of the phagocytes (**Figure S1**, left). Moreover, to confirm the intracellular inhibition of *L. corymbifera* growth, we evaluated the host-cell membrane integrity. For that purpose, we measured the percentage of LDH released from primary monocytes and the monocytic cell line MM6 after exposure to the fungus (**Figure 1F**). After sixteen hours, we observed only a slight increase in LDH release from the infected primary monocytes (13,3 ± 14,37) and the monocytic cell line MM6 (5,74 ± 6,979), indicating that most monocytes remained viable after infection with *L. corymbifera*. To confirm these observations, we stained infected monocytes with Fixable Viability Dye eFluor™ 780 and observed that (91,13 ± 9,413) percent of the phagocytes were viable even after twenty-four hours of infection (**Figure S3B**). Finally, we evaluated the viability of phagocytosed spores by counterstaining non-phagocytosed spores with CFW and using PI to measure viability (**Figure S3A**). We observed that 91,52 ± 6,255 percent of phagocytosed spores were still alive after 24 hours of engulfment (**Figure 1G**). Altogether, our results indicate that internalized spores of *L. corymbifera* remain alive within human monocytes over 24 hours, do not germinate, and inflict minimal damage to the host cell.

### *L. corymbifera* viable spores trigger a pro-inflammatory response by human monocytes

**Figure 2:**
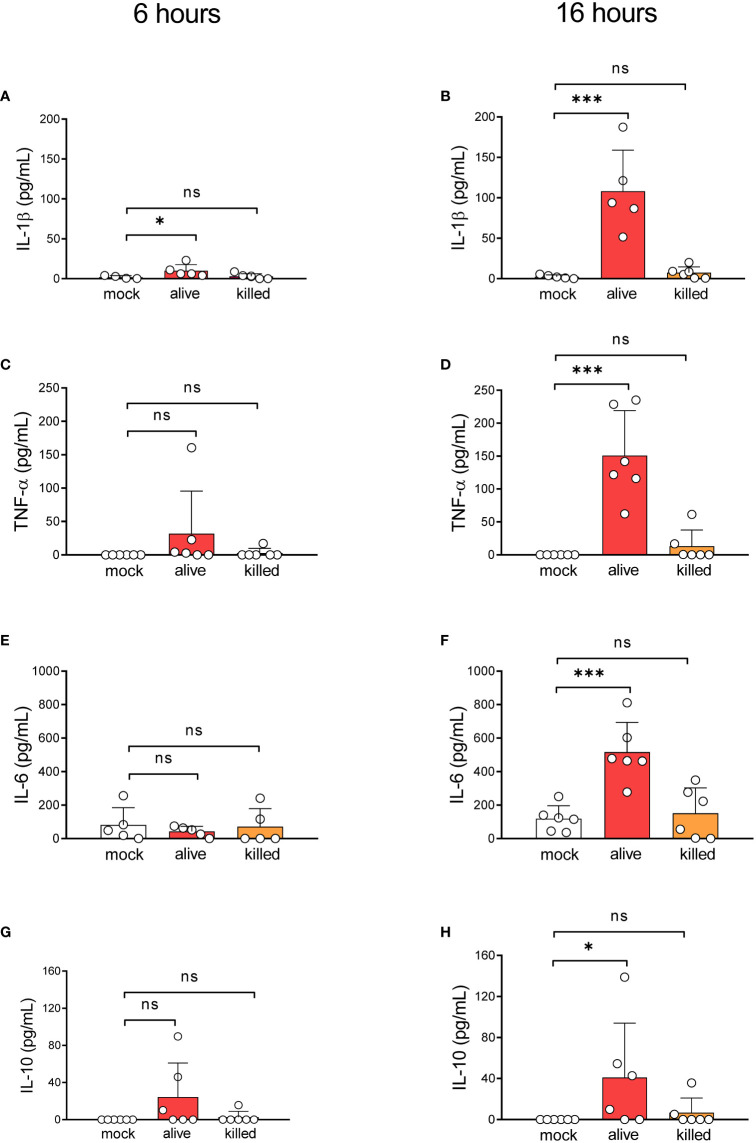
Alive resting spores of *L corymbifera* trigger early inflammatory responses by human monocytes. Pro-inflammatory cytokine release after six **(A, C, E)** and sixteen **(B, D, F)** hours of infection with *L. corymbifera*. **(G, H)** Anti-inflammatory cytokine production after infection with *L. corymbifera*. Bars represent means ± SD of data obtained from three independent experiments, each with one or two different donors, which are represented by dots. Differences among groups were assessed by one-way ANOVA for normally distributed data and Krustal-Wallis for non-normally distributed. *p< 0,05; ***p<0,001. S. proteins indicate surface proteins. ns indicates not significant.

Our previous results indicate that most monocytes remain viable during infection with *L. corymbifera* while restraining the intracellular growth of the fungus. Hence, we wondered whether monocytes could contain the fungal infection while alerting further immune cells *via* cytokine production. Therefore, we quantified the production of the pro-inflammatory cytokines IL-1β, TNF-α, IL-6, and the anti-inflammatory cytokine IL-10 during exposure to *L. corymbifera* (**Figure 2**). We observed that after sixteen hours of infection monocytes produced a significant amount of IL-1β, TNF-α, IL-6 and IL-10 in response to viable fungal spores (**Figures 2B, D, F, H**). While IL-1β, was the only cytokine significantly released after six hours of exposure to the pathogen, in comparison to monocytes without stimuli (**Figure 2A**). Then, we evaluated whether fungal viability could influence the production of these cytokines. For this purpose, we measured cytokine release after infection with PFA-inactivated spores. We observed that cytokine production was significantly impaired after incubation with killed spores, which suggests that activation of the monocytes’ inflammatory response to *L. corymbifera* requires metabolically active spores.

### The virulent strain of *L. corymbifera* generates a broader inflammatory response in human monocytes, compared to the reference strains of *R. delemar* and *M. circinelloides*

**Figure 3:**
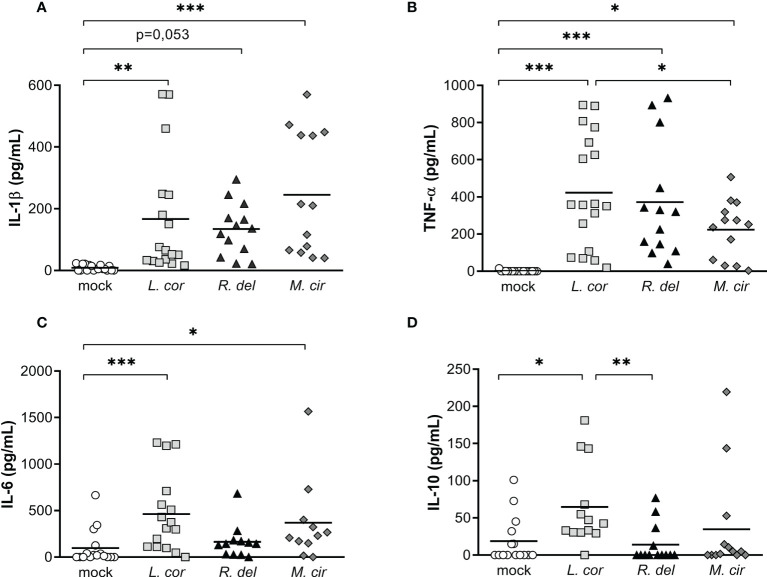
Resting spores of the virulent strain of *L corymbifera* induce a higher production of TNF-α, IL-6, and IL-10 compared tothe clinical reference strains *R. delemar* and *M. circinelloides*. **(A–C)** Monocytes’ pro-inflammatory cytokine and **(D)** anti-inflammatory IL-10 release after sixteen hours of exposure to alive spores of the most common pathogenic Mucorales. Lines represent the mean of data obtained from six independent experiments, each with two or three different donors, which are represented by symbols. Differences among groups were assessed by one-way ANOVA for normally distributed data and Krustal-Wallis for non-normally distributed. *p< 0,05; **p<0,01; ***p<0,001. *L corymbifera* (L.cor), *R. delemar* (R. del), *M. circinelloides* (M.cir).

Most of the reported cases of mucormycosis are caused by *Rhizopus delemar*, *Mucor circinelloides*, and *Lichtheimia corymbifera* species (5, 9). Although infection by these Mucorales shares similar characteristics, previous studies reported unique features that may determine the pathogenicity of each species (43). Hence, we evaluated whether monocytes produce a particular inflammatory response against the most virulent strain of *L. corymbifera* JMRC : FSU:09682 and the clinical reference strains of *R. delemar* 99-880 and *M. circinelloides* CBS277.49. For that purpose, we quantified the production of IL-1β, TNF-α, IL-6, and IL-10 after sixteen hours of exposure to the fungi (**Figure 3**). Despite a broad variation among the donors, we found that *M. circinelloides* and *L. corymbifera* induced significant production of IL-1β, contrary to *R. delemar* which triggered a non-significant but higher release of the cytokine compared to mock-treated monocytes (**Figure 3A**). Moreover, all mucoralean species produced a significant amount of TNF-α compared to the mock-treated monocytes, being *M. circinelloides* the lowest inducer of this cytokine (**Figure 3B**). *L. corymbifera* and *M. circinelloides* exerted production of the pro-inflammatory cytokine IL-6 (**Figure 3C**), and only *L. corymbifera* prompted a significant anti-inflammatory response *via* IL-10 production (**Figure 3D**). Altogether, these results suggest that the mucoralean strains used in this study promote a common signaling inflammatory cascade that results in TNF-α production. However, they may conserve particular traits, such as fungal cell-wall constituents (44), that promote differentcytokines profile. Nonetheless, to consider virulence variability within each species, more strains should be assessed in future studies.

### IL-1β production in response to *L. corymbifera* predominantly dependent on TLR priming

**Figure 4:**
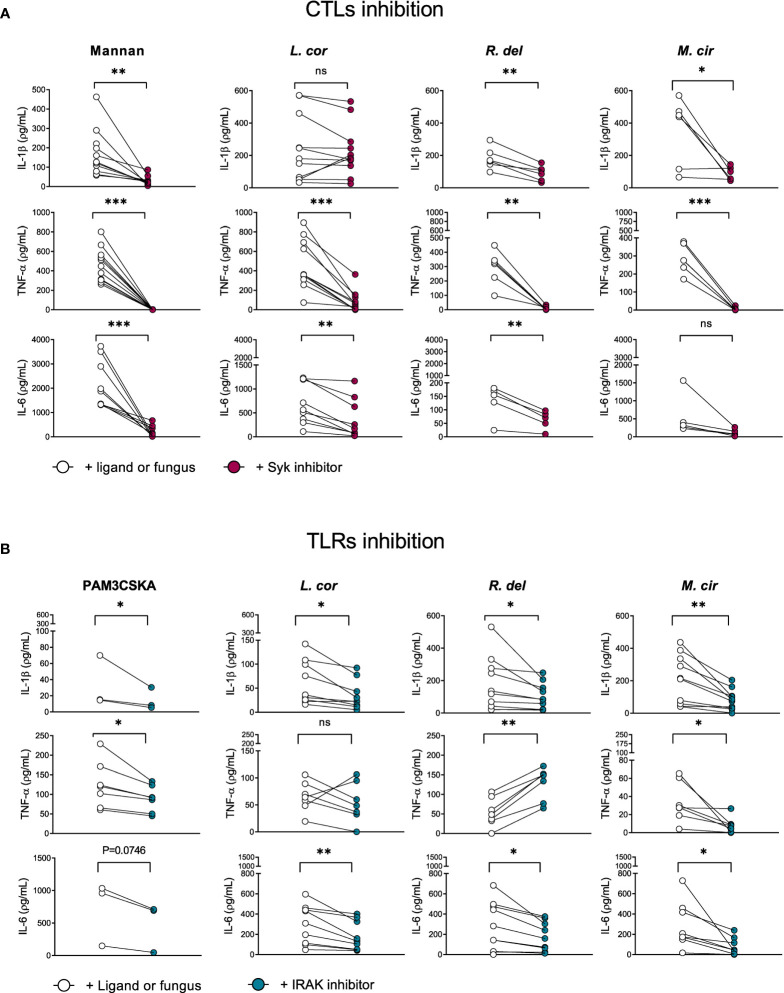
IL-1β production in response to *L corymbifera* is predominantly primed *via* the IRAK signaling pathway in human monocytes. Downstream mediators of TLRs and CTLs were inhibited for three hours before infection with the mucoralean fungi. **(A)** Pro-inflammatory cytokines concentrations after inhibition of the Tyrosine-protein kinase (Syk) or **(B)** the Interleukin-1 Receptor-Associated-Kinase-1/4 (IRAK), During IRAK inhibition PAM3CSKA was used as a specific ligand, whereas mannan was used during Syk inhibition. Dots and lines represent paired values of every single donor. Data was obtained from three independent experiments, each with two or three different donors, and differences among groups were assessed by paired t-test. *p< 0,05; **p<0,01; ***p<0,001. ns indicates not significant. *L corymbifera* (L. cor), *R. delemar* (R. del), *M. circinelloides* (M. cir).

**Figure 5:**
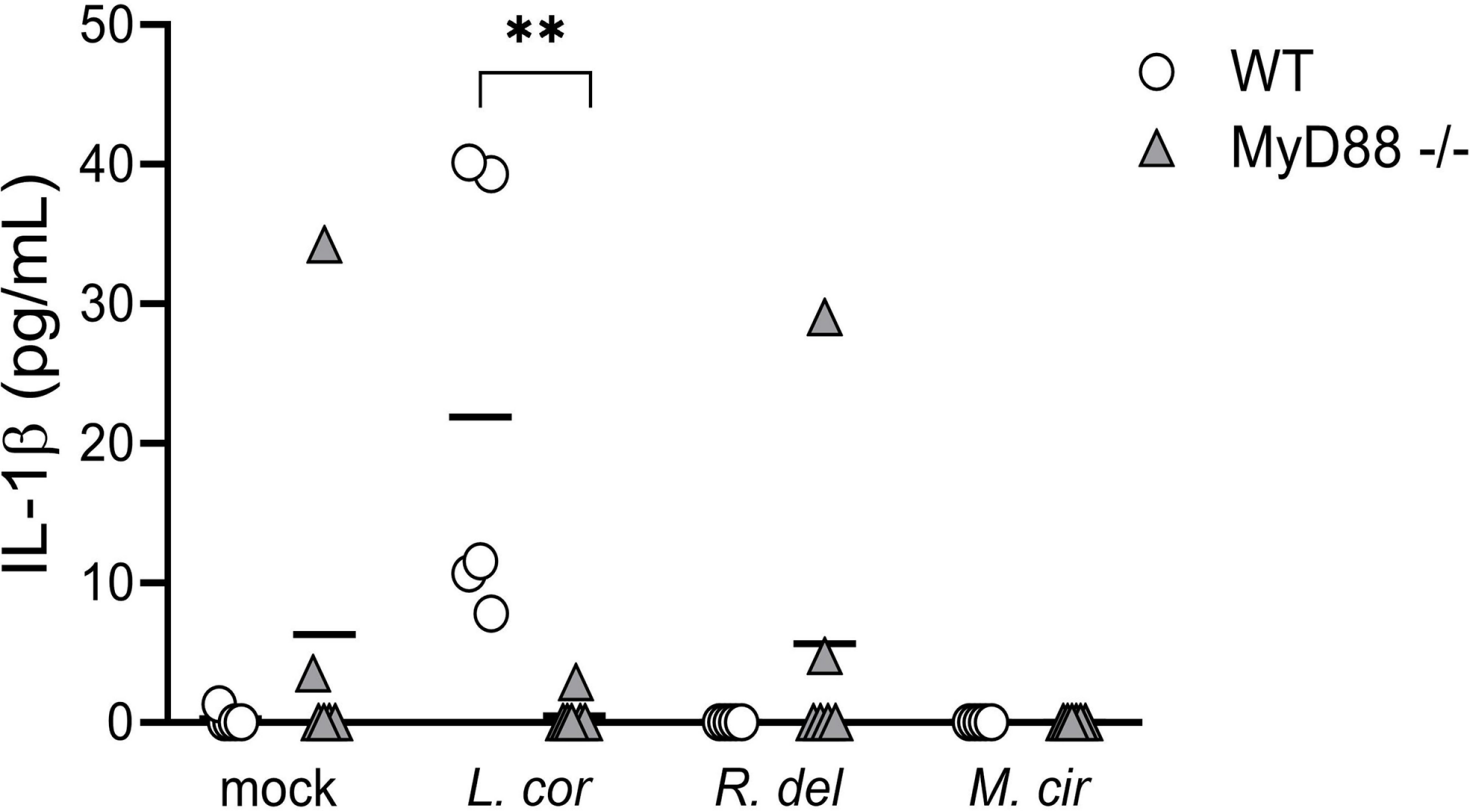
Bone marrow-derived monocytes (BMDM) from MyD88^-/-^ mice failed to induce IL-1β release in response to *L corymbifera.* BMDM lacking the adaptor molecule MyD88 were incubated with the mucoralean fungi for sixteen hours. Subsequently, the production of IL-1β was quantified by ELISA. Lines represent the mean of data obtained in two independent experiments, each with three different mice, which are represented with symbols. Differences among groups were assessed by Krustal-Wallis as non-normally distributed data. **p<0,01.

Cytokines are synthesized upon activation of PRRs through different downstream signaling cascades. These signaling pathways are determined by the type of fungal PAMPs that triggers either the CTLs, TLRs, or both of them for a cooperative amplification of fungal recognition and resolution of the infection (23, 28, 29, 45). Certainly, when evaluating pro-inflammatory cytokine production in response to mucoralean fungi, we observed that each downstream mediator, the Tyrosine-protein kinase (SYK)—a central mediator of the CTLs axis— and the Interleukin-1 Receptor-Associated-Kinase (IRAK), a central effector molecule of TLRs, regulate the inflammatory response by human monocytes. However, TNF-α production against *R. delemar* was reduced after SYK inhibition (**Figure 4A**) and increased after IRAK inhibition (**Figure 4B**), while TNF-α production against *L. corymbifera* was significantly impaired after SYK inhibition (**Figure 4A**) but not to IRAK inhibition. In the case IL-6 production in response to *M. circillenoides*, was significantly reduced to IRAK inhibition but not to SYK inhibition. Furthermore, IL-1β production in response to *L. corymbifera* significantly decreased only after the inhibition of IRAK (**Figure 4B**), but not to SYK (**Figure 4A**), These results suggest a predominant dependency on TLR priming to produce IL-1β in response to *L. corymbifera* infection. To confirm this, we infected BMDMs from mice lacking the adaptor protein MyD88, which regulates the downstream signaling cascade of TLRs, including IRAK (46). As a result, we observed that MyD88 -/- monocytes failed to induce IL-1β after infection with *L. corymbifera* in contrast to WT monocytes (**Figure 5**). Interestingly, our previous results showed that IL-1β was induced in response to *L. corymbifera* during the early and late stages of the infection (**Figures 2A, B**). Moreover, parallel testing of the inhibitors of TLRs, CTLs, and NF-KB revealed no increase in pro-inflammatory cytokine production which provides evidence for a working experimental system (**Figure S4**). Therefore, our results suggest that IL-1β production in response to *L. corymbifera* could be predominantly triggered by TLR priming.

### Inhibition of TLR4 reduced the inflammatory response by monocytes against Mucorales

**Figure 6:**
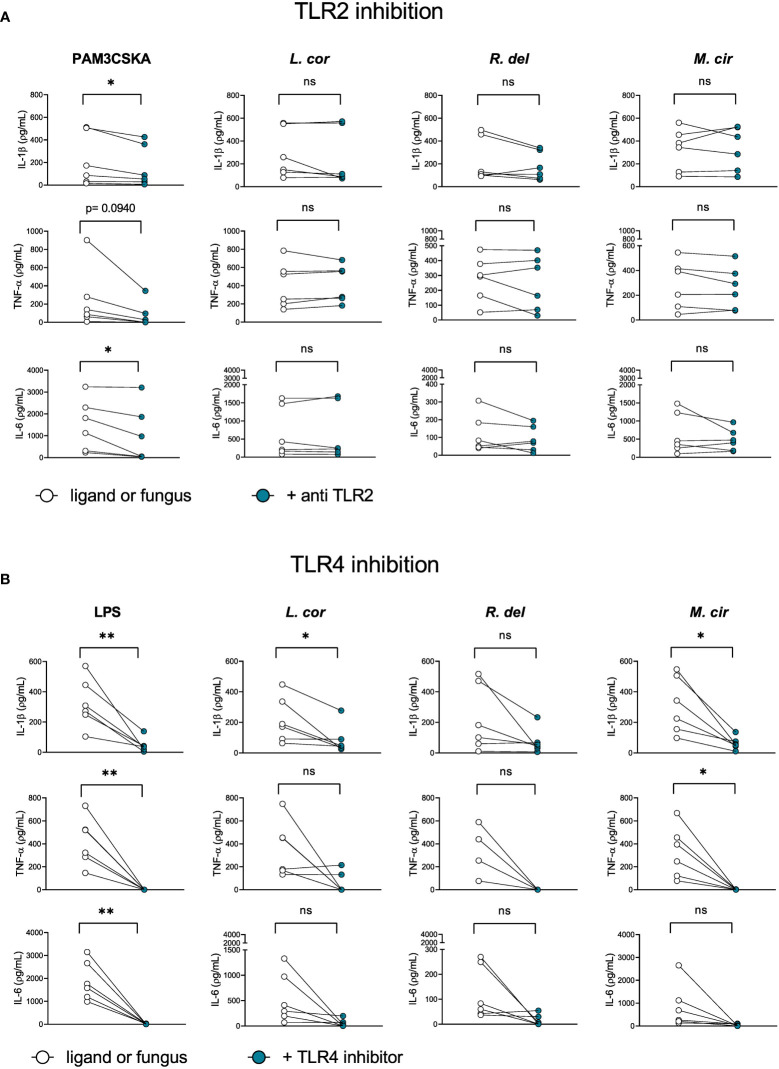
TLR4 contributes to the activation of the inflammatory response by primary human monocytes against*L corymbifera* and *M. circinelloides*. Neutralizing TLR2-antibody and TLR4 inhibitor were incubated for three hours before infection with the Mucorales. **(A)** Pro-inflammatory cytokines concentrations after inhibition of TLR2 or **(B)** TLR4. PAM3CSKA was used as a specific ligand for TLR2 activation and LPS forTLR4 activation. Dots and lines represent paired values of every single donor. Data were obtained from three independent experiments, each with two or three different donors, and differences among groups were assessed by paired t-test. *p< 0,05; **p<0,01. ns indicates not significant. *L corymbifera* (L. cor), *R. delemar* (R. del), *M. circinelloides* (M. cir).

Since the axis of TLRs seems to play an important role in the host-inflammatory response against Mucorales, we evaluated the particular contribution of the receptors TLR2 and TLR4 (**Figure 6**), which are known to mediate antifungal immunity against other fungi, such as *C. albicans* and *C. neoformans* (28, 29, 47, 48). In the case of Mucorales, we observed that TLR2 inhibition did not influence cytokine production in response to Mucorales fungi (**Figure 6A**). On the other hand, TLR4 inhibition promoted a significant reduction of IL-1β after infection with *L. corymbifera* and *M. circinelloides* and TNF-α in response to *M. circinelloides*. (**Figure 6B**), which suggests a predominant role of TLR4 in the activation of the monocyte inflammatory response against these Mucorales, more probably by recognition of a polysaccharide such as a glucan given that it is the major component of the spore cell wall in *M. circinelloides* and play an important role in the immune response against *Rhizopus by* dendritic cells (49, 50). Furthermore, we observed β-1,3-glucans in the early stages of *L. corymbifera* germination (**Figure S5**).

### Inhibition of NF-kB decreases IL-1β, TNF-α, and IL-6 production in response to *L. corymbifera*

**Figure 7:**
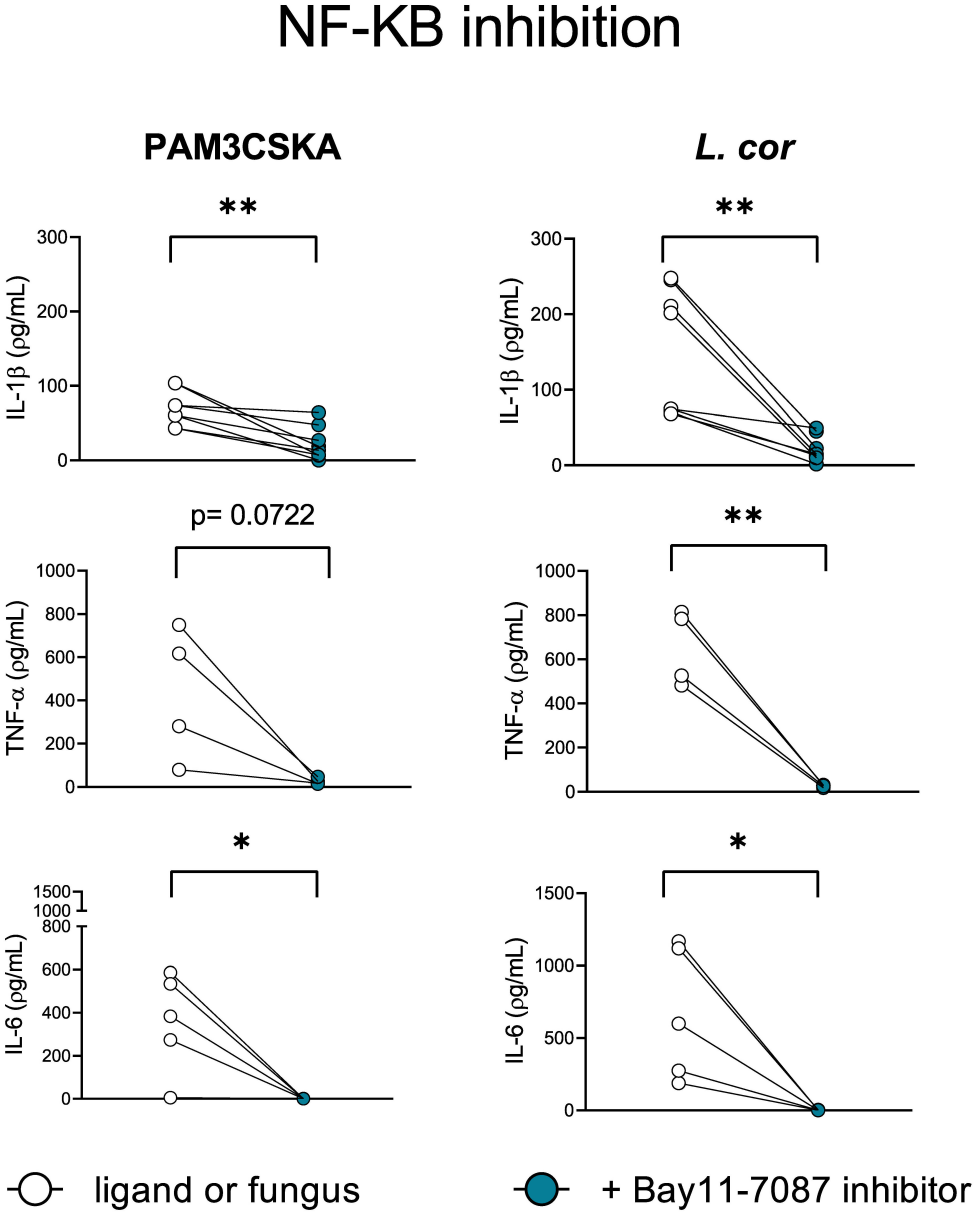
NF-kB is a central mediator of the inflammatory response during infection with *L. corymbifera*. Monocytes were incubated for three hours with the NF-KB inhibitor (Bay11-7087), before infection with *L. corymbifera* for sixteen hours. Pro-inflammatory cytokines were measured by ELISA. Dots and lines represent paired values of every single donor. Data were obtained from three independent experiments, each with two or three different donors, and differences among groups were assessed by paired t-test. *p< 0,05; **p<0,01. *L corymbifera* (L. cor).

Previous studies described the transcription factor NF-kB as a pivotal mediator of the inflammatory response. During infection, immune cell recognition of diverse PAMPs and DAMPs by PRRs activates the NF-kB canonical pathway and induces the transcription of pro-inflammatory genes encoding inflammatory mediators (51). Thus, we evaluated the contribution of the NF-kB canonical signaling pathway to the induction of pro-inflammatory cytokines by preventing its translocation into the nucleus with the inhibitor BAY11-7082 (**Figure 7**, **Figure S4**). Certainly, IL-1β, TNF-α, and IL-6 production were significantly reduced after inhibition of NF-kB when infected with *L. corymbifera* (**Figure 7**), suggesting the existence of a downstream convergence for these cytokines signaling pathways. Furthermore, inhibition of the canonical NF-kB pathway did not completely abolish IL-1β release in contrast to TNF-α and IL-6 production (**Figure 7**), suggesting that inflammasome-independent mechanism, as previously described by (52) could be contributing to process pro-IL-1b into IL-1β by human monocytes in response to *L. corymbifera*.

### NF-kB modulates monocytes apoptosis during infection with *L. corymbifera*

**Figure 8:**
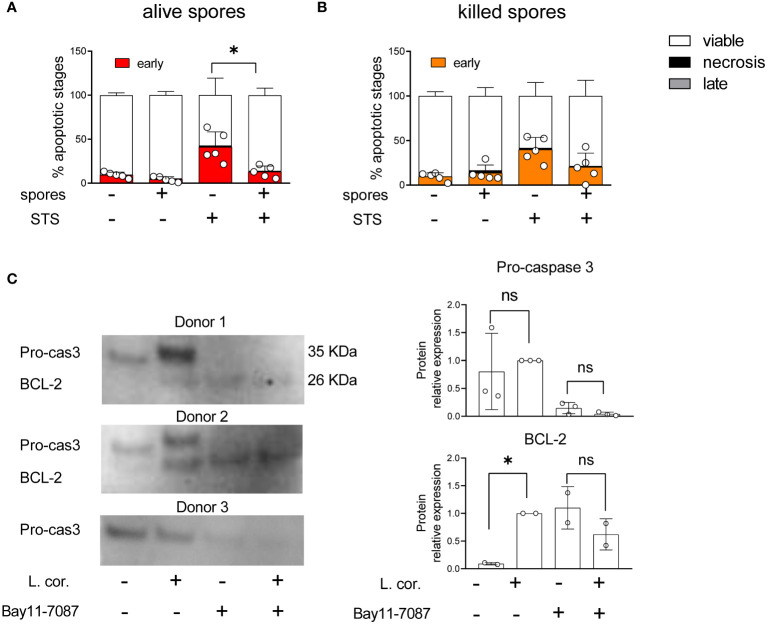
Viable spores of *L corymbifera* reduce early apoptosis in monocytes and trigger survival response in human monocytes. **(A, B)**. After phagocytosis of fungal spores, human monocytes were exposed to staurosporine (STS), a kinase inhibitor that induces apoptosis. Subsequently, monocyte apoptotic stages were evaluated by flow cytometry. **(C)** Detection of pro-caspase 3 (Pro-cas3) and the anti-apoptotic protein BCL-2 after exposure to Bay11-7087, an NF-KB inhibitor, and/or resting spores of *L corymbifera*. Bars represent means ± SD of data obtained from at least three independent experiments. Differences among groups were assessed by one-way ANOVA. *p< 0,05. ns indicates not significant.

**Figure 9:**
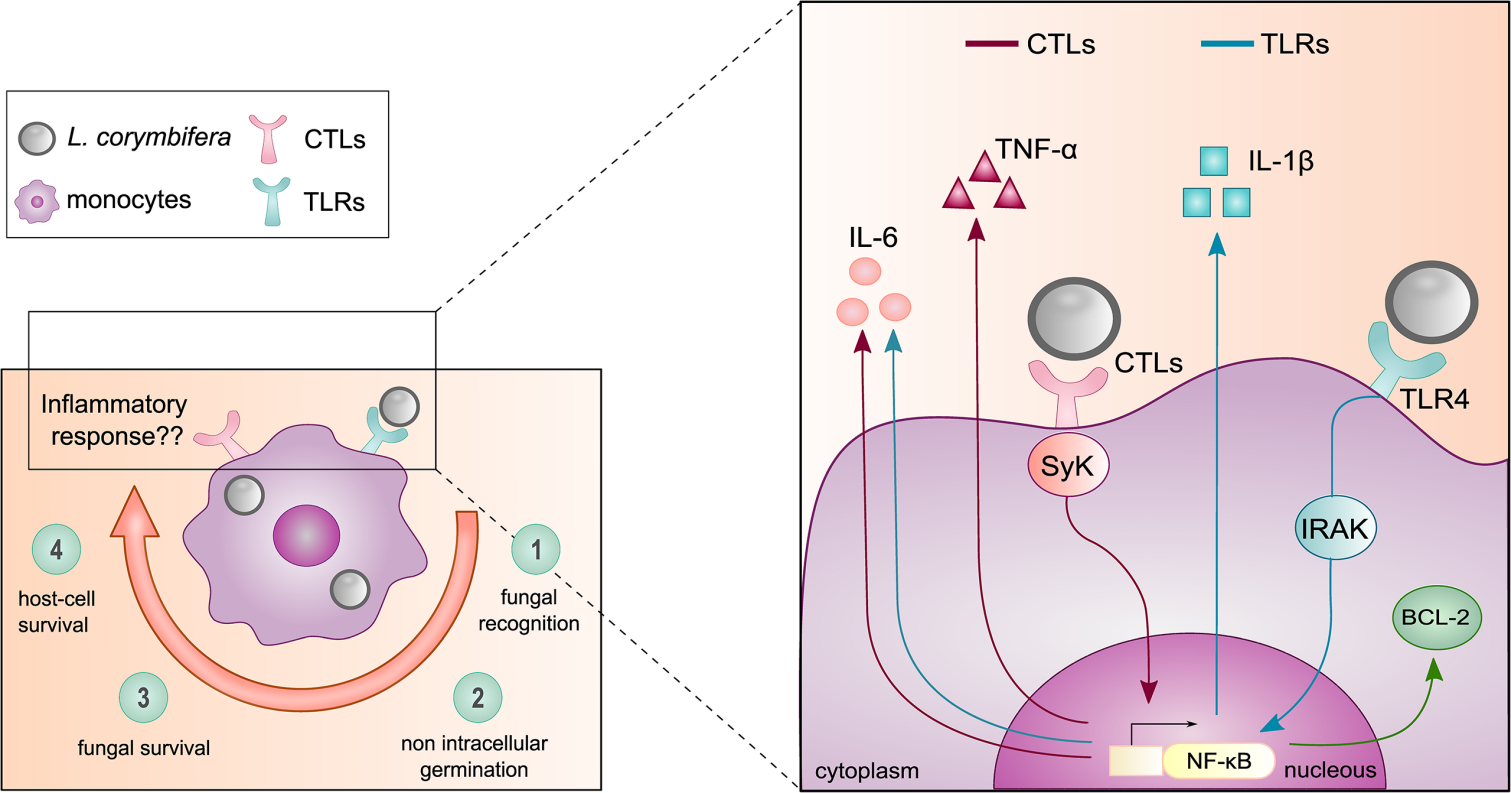
Mechanism of the monocyte inflammatory response against the pathogenic fungus *L. corymbifera*. (*Left panel*) Main findings of the initial host-pathogen interactions between human monocytes and *L. corymbifera* spores over infection. Clockwise, 1) Recognition of the fungal spores within the first hours of infection. 2,3) intracellular growth inhibition and fungal survival after 24 hours post-infection. 4) Minimal damage to the host by the fungus. (*Right panel*) Monocytic pro-inflammatory cytokine activation in response to *L. corymbifera*. The production of the pro-inflammatory cytokine IL-1β is predominantly induced by TLRs (blue arrows), especially *via* TLR4. Whereas, TNF-α release is mediated by CTLs (red arrows), and IL-6 by synergistic activation of CTLs and TLRs (blue arrows). These signaling pathways are regulated by the transcription factor NF-κB, which also modulates the apoptotic fate of infected human monocytes *by* promoting the expression of the anti-apoptotic protein BCL-2 (green arrow).

The downstream signaling and release of IL-1β by primary monocytes depend on the intensity of the pro-inflammatory stimuli. Whereas only TLR4 activation induces slow IL-1β release, simultaneous stimulation of several TLRs promotes faster IL-1β secretion and high levels of Reactive Oxygen Species (ROS), which are instrumental for antifungal immunity (23, 27, 40). Accordingly, single monocyte stimulation induces apoptosis, while diverse stimuli trigger pyroptosis (23). Our previous results suggest that *L. corymbifera* have a long-term intracellular survival (**Figures 1F, H**), do not cause significant damage in monocytes (**Figure 1G** and **Figure S2C**), and induce the early release of IL-1β (**Figures 2A, B**) predominantly primed by TLR4 activation (**Figure 6B**). All these findings led us to evaluate whether *L. corymbifera* could influence the monocyte’s apoptotic response. For that purpose, we infected human monocytes with spores and induced apoptosis with STS, a well-known chemical inducer of apoptosis by activation of caspase-3 (53, 54) (**Figure 9**). Surprisingly, *L. corymbifera* did not induce apoptosis in monocytes but did significantly reduce early apoptosis in the cells treated with STS when fully alive.(**Figures 8A, B**, **Figure S6**), similar to our results of IL-1β production (**Figures 2A, B**), suggesting that intracellular viability of *L. corymbifera* spores could have an influence on both, the inflammatory response and the apoptotic fate of monocytes. We further exposed human monocytes to the NF-kB inhibitor BAY11-7082, as well as *L. corymbifera* spores, and evaluated the expression of the pro-caspase 3, which is cleaved during apoptosis, and the anti-apoptotic protein BCL-2, which exerts a survival response to several apoptotic stimuli. As a result, the pro-caspase 3 protein expression decreased in the presence of the inhibitor but not of the spores (**Figure 8C**), whereas BCL-2 protein expression increased after exposure to spores, as well as BAY11-7082. These results together suggest that *L. corymbifera* spores do not induce apoptosis but a survival response in monocytes which might impact on the release of cytokines and the outcome of the infection.

## Discussion

Fungal clearance predominantly depends on the recognition and intracellular killing of pathogens by professional phagocytes. However, some pathogenic fungi such as *A. fumigatus*, *C. neoformans*, and *C*. *albicans* have developed different strategies to evade immune detection either *via* modification in their cell-wall constituents or secretion of inhibitory fungal molecules (28, 40, 55–59). In contrast to the previous pathogenic fungi, the most virulent strain of *L. corymbifera* (FSU: 009682) did not exhibit avoidance of immune detection by monocytes and neutrophils since its sporangiospores were rapidly and efficiently phagocytized by both cell types (**Figure 1A**), which suggests the presence of highly immunogenic molecules in its cell wall, such as β-1,3-glucans, as shown in *L. corymbifera* germlings (**Figure S5**), or unmasked chitosan after removal of glucans (60). However, recognition of an attenuated strain of *L. corymbifera* (FSU:10164) was reduced approximately by half when compared to the virulent strain (**Figure S1B**), indicating possible differences in the cell wall composition of each strain, which could influence on their virulence. Nonetheless, given the clinical relevance of *L. corymbifera* (FSU: 009682), we studied the monocyte response to this particular strain.

In contrast to several pathogenic fungi that avoid intracellular killing by lysing the host’s cells (28, 40), *L. corymbifera* did not cause significant damage in phagocytic monocytes (**Figure 1F**) and did not impair their viability over 24 hours of infection (**Figure S3B**), which was unexpected considering that several clinical cases of mucormycosis reported rapid destruction of tissue and necrosis, as well as the epithelial and endothelial damage caused by other mucoralean fungi upon intracellular growth (1, 6, 30, 61, 62). Moreover, *L. corymbifera* spores did not exhibit intracellular germination and remained alive after 24 hours of phagocytosis by human monocytes (**Figures 1D, E, G**), suggesting that the fungus inhibits the intracellular enzymatic degradation, which occurs in the phagolysosome after activation of specific PRRs and recruitment of specific phagosome proteins (28, 63).

In addition to pathogen recognition and clearance, the immune response also involves the production of cytokines (14, 15). These proteins induce inflammation as an “alarm system” to amplify the antifungal response and recruit several phagocytes to the site of infection, (14, 64). For instance, IL-1β promotes further inflammatory proteins, hematopoiesis, differentiation of T_H_ cells as well as resolution of the inflammation *via* IL-10 induction (15, 21–23), whereas TNF-α mediates the host-apoptotic responses and the inflammatory response, and IL-6 promotes trafficking of leukocytes, production of acute-phase proteins, cell proliferation, and survival (24, 25). In this study, we observed that upon phagocytosis of *L. corymbifera*, human monocytes released IL-1β during the first hours of infection, while TNF-α and IL-6 were significantly released in later stages of the infection (**Figure 2**). Our observations suggest that IL-1β has an important role in antifungal immunity against *L. corymbifera* by promoting an early response in monocytes, which may alert other immune cells and induce further pro-inflammatory cytokines as observed in previous studies (22, 23, 36, 65). Interestingly, only viable spores of *L. corymbifera* induced pro-inflammatory cytokines compared to killed spores. This finding suggests that inflammatory response to the fungus may rely on a dynamic fungal cellular process, such as re-arrangement of the fungal cell wall or secretion of fungal molecules, as previously observed in *R. delemar* with the secretion of the ricin-like toxin Mucoricin (66).

During fungal infection, any delay in the activation of the inflammatory response and recruitment of phagocytes to the site of infection could promote faster fungal dissemination and destruction of the tissue (67, 68). Following this, previous studies reported that patients with invasive pulmonary mucormycosis presented extensive angioinvasion but a limited or moderate inflammatory response (69–71). Therefore, we investigated if the production of inflammatory cytokine was repressed in human monocytes exposed to common pathogenic mucoralean fungi. Interestingly, we observed that virulent strain of *L. corymbifera* induced a broader inflammatory response in human monocytes compared to the reference strains, *Rhizopus delemar* 99-880 and *Mucor circinelloides* CBS277.49, which are the most reported strains in previous host-pathogen interaction studies (30, 34, 72–75)(**Figure 3**). For this reason, we postulate that the virulent strain of *L. corymbifera* is a good candidate to elucidate molecular mechanisms, such as priming of PRRs and cytokine signaling pathways activated during infection with Mucorales, since it may possess unique traits, either cell-wall constituents or secreted fungal molecules that promote a faster and broader inflammatory response compared to the other strains.

In this study, we provide new insights into candidate signaling pathways involved in the inflammatory response against Mucorales by evaluating the production of common pro-inflammatory cytokines upon inhibition of the main PRRs’ downstream mediators, such as the Tyrosine-protein kinase (SYK) that modulates the CTLs signaling pathway, or the Interleukin-1 Receptor-Associated Kinase (IRAK) that mediates signaling from most of the TLRs (26, 28, 29). We observed that both, SYK and IRAK inhibition resulted in a significant reduction of IL-6 and TNF-α release during infection in response to the mucoralean species (**Figures 4A, B**), which is in agreement with previous studies that reported cooperation between CTLs and TLRs to amplify the inflammatory immune response (28, 45, 76). However, we observed that production of IL-6 and TNF-α depending on a single-axis activation *via* CTLs, or TLRs relies on the type of mucoralean fungi. As an example, we observed a predominant modulation of IL-6 *via* TLRs after infection with *M. circinelloides*, and TNF-α production *via* CTLs after infection with *R. delemar* (**Figure 4A**). Curiously, TNF-α expression in response to *R. delemar* was increased after IRAK inhibition. Given that the inhibitor alone did not influence its production (**Figure S4B**), we hypothesize that TNF-α is overproduced *via* CTLs as a compensatory effect. Interestingly, the use of TNF antagonists as treatment for autoimmune diseases has been associated with an increased incidence of fungal infections (77, 78). IL-1β production in response to *L. corymbifera* was significantly reduced only after IRAK inhibition (**Figure 4B**), suggesting a specific route to produce this particular cytokine. This finding may provide new insights for future therapies since IL-1β is produced early in response to *L. corymbifera.* Thus, a balanced enhancement of this cytokine may promote higher phagocyte recruitment and prevent further fungal invasion. Nonetheless, potential benefits must be weighed against possible complications to avoid detrimental hyperinflammatory responses.

Among the TLRs, TLR2 and TLR4 have been extensively studied in the immune response against other pathogenic fungi. These receptors recognize specific fungal PAMPs, such as α-1,4-glucans, Glucuronoxylomannans, and Phospholipomannans in the case of the TLR2; and O-linked mannans and Rhamnomannansin in the case of TLR4 (28, 29, 47, 48), which later on leads to the nuclear translocation of the central regulator NF-κB (51, 79). In that regard, we observed that TLR4 inhibition reduced the production of TNF-α in response to *M. circinelloides* and IL-1β secretion after exposure to *L. corymbifera* and *M. circinelloides* (**Figure 6B**), suggesting that TLR4 has a predominant role in the activation of these signaling pathways. Furthermore, TLR2 inhibition did not affect the pro-inflammatory cytokine production after exposure to the Mucorales. A previous study by Chamilos et al. showed that exposure to *R. delemar* induced tlr2 expression (80). However, the authors measured the mRNA levels in human polymorphonuclear cells instead of total proteins in monocytes as we described here, potentially explaining the discrepancy between studies. Moreover, we observed that NF-κB inhibition reduced IL-1β production and completely abolished TNF-α and IL-6 (**Figure 7**), suggesting that IL-1β production partially depends on the activation of the transcription factor, while TNF-α and IL-6 pathways converge downstream in NF-κB. Altogether, our findings suggest that each mucoralean species possesses unique cell-wall constituents that may activate different TLRs and CLRs and induce the production of particular pro-inflammatory cytokines, most of them regulated by NF-κB. Hence, future studies should focus on the identification of these specific receptors and their ligands.

Previous studies described a close connection between cell death mechanisms and inflammation during infection, in which the type of activated caspases, produced cytokines, and intensity of the initial pro-inflammatory stimuli determine the nature of the subsequent inflammatory mechanism and the types of programmed cell death (79, 81). As an example, apoptotic responses induce the production of IL-1β, while necroptosis causes the release of IL-1α, in both cases, the cytokines lead to the activation of downstream inflammatory central regulators, such as the transcription factor NF-κB (23, 51, 79). In that regard, we observed that alive spores of *L. corymbifera* reduced early apoptosis in human monocytes contrary to killed spores (**Figure 8A**), suggesting that the apoptotic fate of monocytes could depend on a dynamic fungal process, similar to our previous observations of IL-1β secretion in response to *L. corymbifera* (**Figure 2B**). Furthermore, we confirmed a connection between the apoptotic and inflammatory responses after detecting pro-caspase 3 expression—a hallmark of apoptosis—in response to the fungus and the NF-KB inhibitor, Bay11-7087. Here, exposure to the fungal spores did not reduce the expression of pro-caspase 3 contrary to the Bay11-7087 treatment that completely abolished the signal (**Figure 8C**). Moreover, monocytes infected with the fungus increased their expression of the anti-apoptotic protein BCL-2 similarly to those exposed to Bay11-7087, suggesting that *L. corymbifera* may influence the apoptotic fate of monocytes by promoting their survival *via* NF-KB modulation. Therefore, we hypothesize that several immune responses to *L. corymbifera* such as cell death and inflammation could be modulated by the family of inducible transcription factors NF-κB.

## Conclusion

Collectively our findings highlight a long-term survival of *L. corymbifera* within human monocytes, as well as resistance to intracellular killing. This trait may confer the fungus a temporal niche to escape the host immune response. Hence, we believe that further immunotherapy-based strategies are needed to enhance the intracellular degradation of the pathogen. In this study, we found that IL-1β plays an instrumental role in antifungal immunity against *L. corymbifera* by promoting an early inflammatory response by monocytes (**Figure 9**). Interestingly, IL-1β is well-known for its key role in the activation and amplification of inflammatory signaling cascades, recruitment of other immune cells to the site of infection, and enhancement of intracellular killing. Here, we provide a predominant route for the production of IL-1β *via* activation of the TLRs-IRAK-NF-κB axis in response to *L. corymbifera*, from which TLR4 is a major PRR involved in the recognition of the fungus. Furthermore, we describe the regulation of TNF-α by the CTLs axis, and IL-6 modulation by both, TLRs and CTLs (**Figure 9**). Altogether, our findings may serve as background to further studies on the inflammatory response driven by the interaction among specific mucoralean ligands and TLR4.

## Data availability statement

The raw data supporting the conclusions of this article will be made available by the authors, without undue reservation.

## Ethics statement

The studies involving human participants were reviewed and approved by the Institute for Transfusion Medicine, Jena University Hospital, after written informed consent of the healthy donors in accordance with ethics committee approval 4357-03/15 and with the Declaration of Helsinki 1975, as revised in 2008. The patients/participants provided their written informed consent to participate in this study. The animal study was reviewed and approved by Thueringer Landesamt für Verbraucherschutz (breeding license 02-052/16). Written informed consent was obtained from the owners for the participation of their animals in this study.

## Author contributions

DM designed and executed the experiments for this study, analyzed the obtained data, and wrote the manuscript. KV conceptualized and supervised the study as well as reviewed and edited the manuscript. SH and ML-T designed the phagocytosis quantification method, provided scientific advice on the study, and supplied human biological material. MW, RA, and BJ designed and performed Murine Bone Marrow-Derived Monocytes co-infection experiments. All authors contributed to the article and approved the submitted version.

## Funding

This work was supported by the German Research Foundation (DFG) through the CRC/TRR 124 Pathogenic fungi and their human host: Networks of interaction (FungiNet), DFG project number 210879364, Projects A1, A6 and C4 to MvLT, KV and BJ, respectively. We thank financial support also from the Leibniz Institute for Natural Product Research and Infection Biology Jena – Hans Knöll Institute (HKI) and the Friedrich-Schiller University (FSU) of Jena.

## Acknowledgments

We are thankful to the International Leibniz Research School (ILRS) and Dr. Christine Vogler for administrative support. to Dr. Hans-Martin Dahse for providing the human monocytes Mono Mac 6 (MM6) cell-line and cell culture material. We are also grateful to Wolfgang Vivas for the critical reading of the manuscript. We thank Caroline Semm (FSU Jena) for technical assistance with strain maintenance. We also express our gratitude to Silke Steinbach for technical support with Western blotting.

## Conflict of interest

The authors declare that the research was conducted in the absence of any commercial or financial relationships that could be construed as a potential conflict of interest.

## Publisher’s note

All claims expressed in this article are solely those of the authors and do not necessarily represent those of their affiliated organizations, or those of the publisher, the editors and the reviewers. Any product that may be evaluated in this article, or claim that may be made by its manufacturer, is not guaranteed or endorsed by the publisher.

## Supplementary material

Supplementary Figure 1
*L. corymbifera* spores do not germinate after fixation with paraformaldehyde (PF) **(A)**. *L. corymbifera* treated-spores after sixteen hours of incubation in RPMI-FCS supplemented medium. Scale bar 20 µm. **(B)** Phagocytosis percentages by primary human monocytes and neutrophils of a virulent (FSU: 9682) and an attenuated (FSU: 10164) strain of *L. corymbifera*. Symbols and bars represent means ± SD of four independent experiments.

Supplementary Figure 2 Gating strategy to quantify percentages of phagocytosis. Isolated human leukocytes were incubated with FITC-labeled spores over 180 minutes, followed by identification with CD45+, while monocytes were gated from CD14+ and neutrophils from CD15+ populations. Phagocytosed spores were identified as FITC+/APC-anti FITC- and adherent spores as FITC+/APC-anti FITC+. Numbers in the gating represent percentages of each population from one typical experiment.

Supplementary Figure 3 Cell viability during fungal infection. **(A)** Gating strategy to determine the viability of phagocytosed spores after 24 hours of co-infection. Calcofluor white (CFW) was used to discriminate between phagocytosed and adherent spores, and Propidium Iodide (PI) for their viability. **(B)** Viability of human monocytes over 24 hours of infection with *L corymbifera* spores. Percentages of viability were determined by staining with Fixable Viability Dye - eFluor™ 780 APC-Cy7 and analyzed by flow cytometry. Bars represent means ± SD of at least three independent experiments.

Supplementary Figure 4 Inhibitors of TLRs, CLRs, and NF-KB do not increase pro-inflammatory cytokine production in absence of spores. Human monocytes were incubated for sixteen hours with the Interleukin-1 Receptor-Associated-Kinase-1/4 inhibitor (IRAK inh.), the Tyrosine-protein kinase inhibitor (Syk inh.), and the NF-KB inhibitor (Bay11-7087). **(A)** IL-1β, **(B)** TNF-α, and **(C)** IL-6 production after incubation with the inhibitors were measured by ELISA. Bars represent means ± SD of data obtained from at least three independent experiments, each with two or three different donors.

Supplementary Figure 5 The initial stage of germination exposed 1,3-β-glucans of *L. corymbifera*. Resting spores of *L. corymbifera* were grown in RPMI cell-culture medium for four hours at 37°C. Germlings and resting spores of *L. corymbifera* were incubated with 1,3- β- glucans, anti-mouse IgG: Alexa 488, and visualized by Confocal Laser Scanning Microscopy. Scale bar represents 10µm distance.

Supplementary Figure 6 Gating strategy to determine apoptosis in human primary monocytes. Annexin V conjugated to Pacific Blue was used to identify apoptotic monocytes and Fixable Viability Dye eFluor™ 780-APC-Cy7 to discriminate between late apoptotic and necrotic cells. Viable cells were identified as Pacific Blue -/APC-Cy7 -, early apoptotic cells as Pacific Blue +/APC-Cy7 -, late apoptotic cells as Pacific Blue +/APC-Cy7 +, and necrotic cells as Pacific Blue -/APC-Cy7 +. Numbers in the gating represent percentages of each population from one typical experiment.

## Abbreviations

Pathogen-Associated Molecular Patterns (PAMPs), damage-associated molecular patterns (DAMPs), Pattern-Recognition Receptors (PRRs), Toll-like receptors (TLRs), C-type lectin receptors (CTLs), NOD-like receptors (NLRs), phosphate-buffered saline (PBS), para-formaldehyde (PFA), fetal bovine serum (FBS), Peripheral Blood Mononuclear Cells (PBMC), Calcofluor-white (CFW), Fluorescein Isothiocyanate (FITC), Staurosporine (STS).
