# Supplementary figures and images for: The TLR-NF-kB axis contributes to the monocytic inflammatory response against a virulent strain of Lichtheimia corymbifera, a causative agent of invasive mucormycosis
Source: Front Immunol. 2022 Oct 13;13:882921. doi: 10.3389/fimmu.2022.882921 (PMC9608459; doi:10.3389/fimmu.2022.882921)

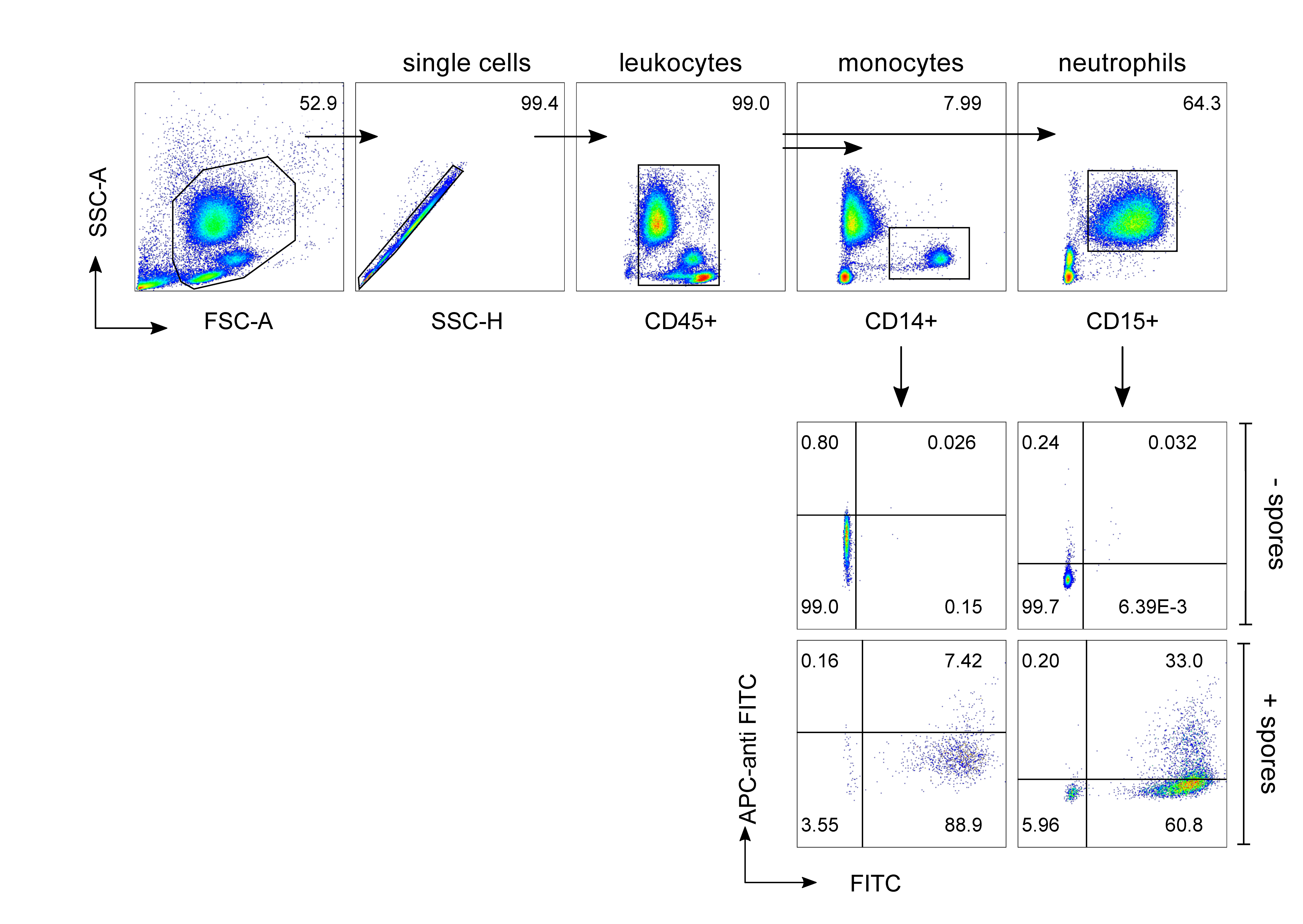

Supplement: Supplementary Figure 2 — Gating strategy to quantify percentages of phagocytosis. Isolated human leukocytes were incubated with FITC-labeled spores over 180 minutes, followed by identification with CD45+, while monocytes were gated from CD14+ and neutrophils from CD15+ populations. Phagocytosed spores were identified as FITC+/APC-anti FITC- and adherent spores as FITC+/APC-anti FITC+. Numbers in the gating represent percentages of each population from one typical experiment. [file Image_2.tiff]

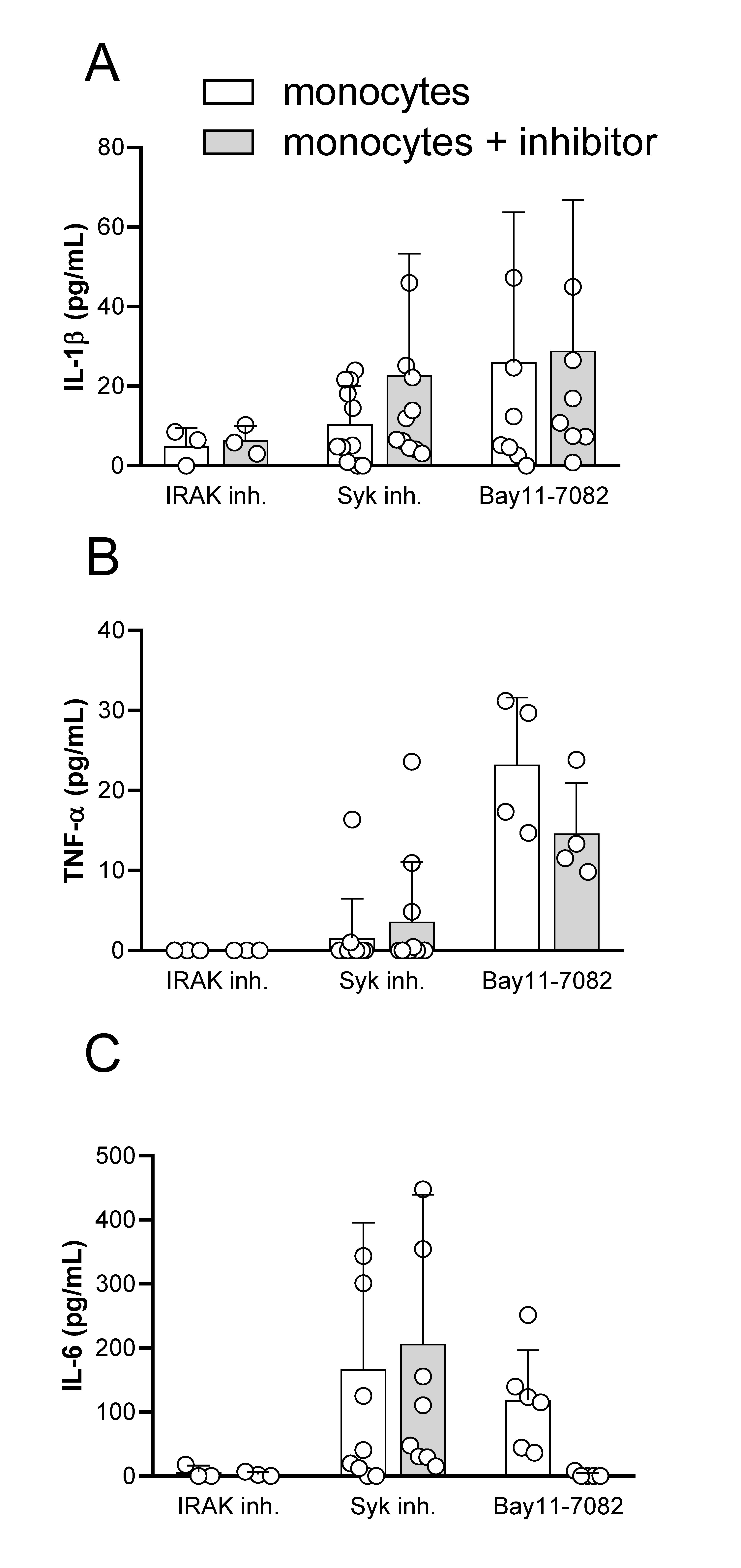

Supplement: Supplementary Figure 4 — Inhibitors of TLRs, CLRs, and NF-KB do not increase pro-inflammatory cytokine production in absence of spores. Human monocytes were incubated for sixteen hours with the Interleukin-1 Receptor-Associated-Kinase-1/4 inhibitor (IRAK inh.), the Tyrosine-protein kinase inhibitor (Syk inh.), and the NF-KB inhibitor (Bay11-7087). (A) IL-1β, (B) TNF-α, and (C) IL-6 production after incubation with the inhibitors were measured by ELISA. Bars represent means ± SD of data obtained from at least three independent experiments, each with two or three different donors. [file Image_4.tif]

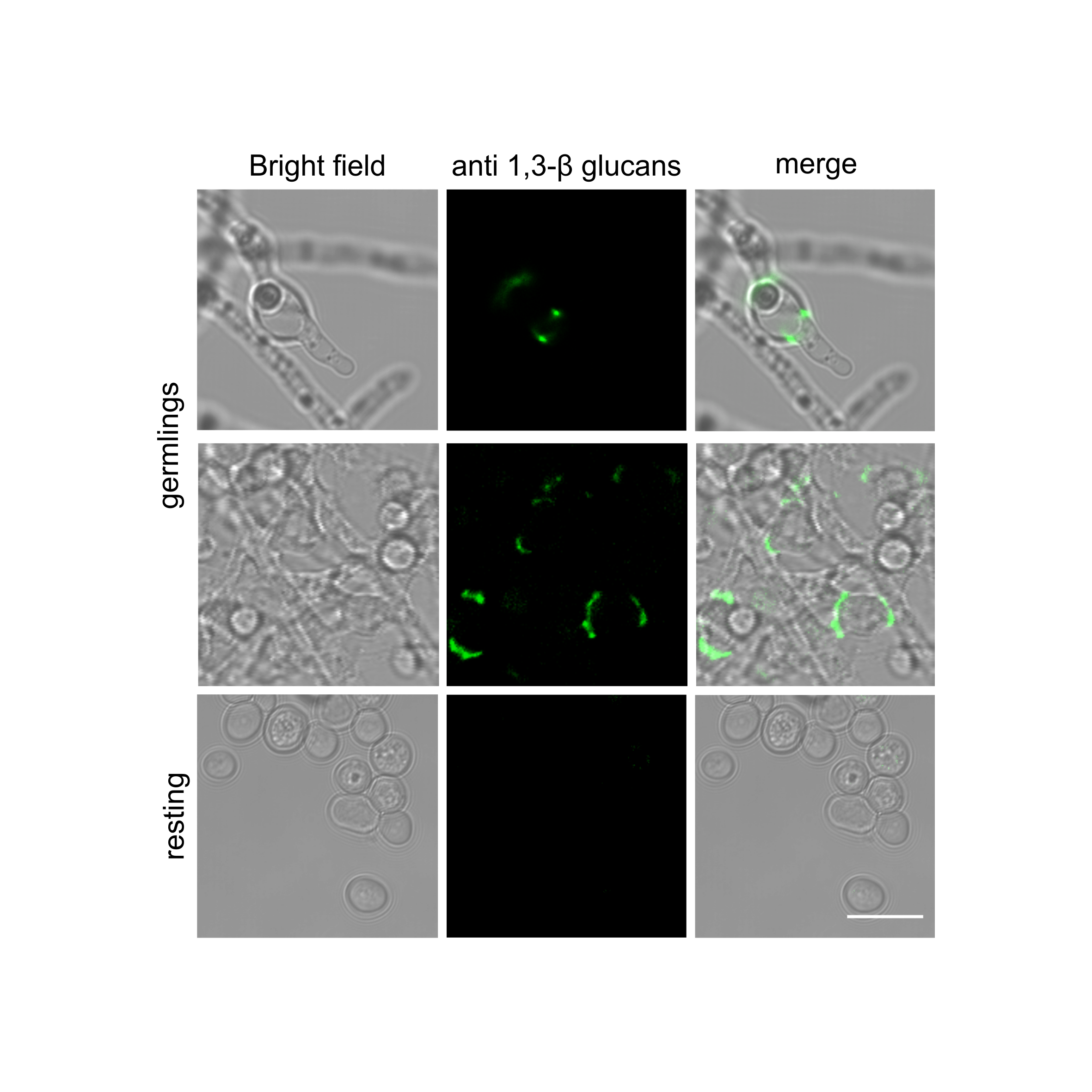

Supplement: Supplementary Figure 5 — The initial stage of germination exposed 1,3-β-glucans of L. corymbifera. Resting spores of L. corymbifera were grown in RPMI cell-culture medium for four hours at 37°C. Germlings and resting spores of L. corymbifera were incubated with 1,3- β- glucans, anti-mouse IgG: Alexa 488, and visualized by Confocal Laser Scanning Microscopy. Scale bar represents 10µm distance. [file Image_5.tiff]

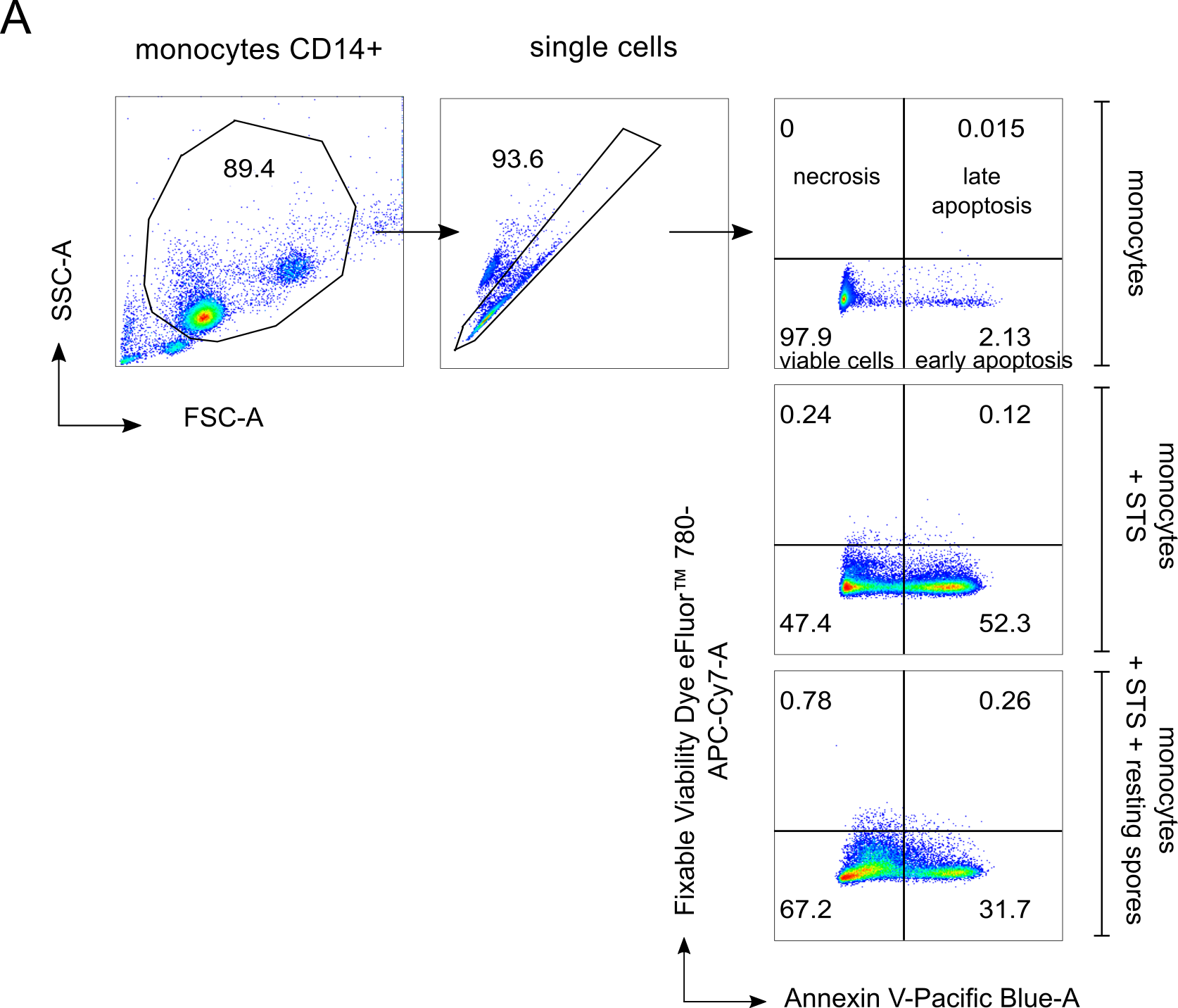

Supplement: Supplementary Figure 6 — Gating strategy to determine apoptosis in human primary monocytes. Annexin V conjugated to Pacific Blue was used to identify apoptotic monocytes and Fixable Viability Dye eFluor™ 780-APC-Cy7 to discriminate between late apoptotic and necrotic cells. Viable cells were identified as Pacific Blue -/APC-Cy7 -, early apoptotic cells as Pacific Blue +/APC-Cy7 -, late apoptotic cells as Pacific Blue +/APC-Cy7 +, and necrotic cells as Pacific Blue -/APC-Cy7 +. Numbers in the gating represent percentages of each population from one typical experiment. [file Image_6.tiff]
